# Reduced inflammation accompanies diminished myelin damage and repair in the NG2 null mouse spinal cord

**DOI:** 10.1186/1742-2094-8-158

**Published:** 2011-11-13

**Authors:** Karolina Kucharova, Yunchao Chang, Andrej Boor, Voon Wee Yong, William B Stallcup

**Affiliations:** 1 Sanford-Burnham Medical Research Institute, La Jolla, CA 92037, USA; 2 St Jude Children's Research Hospital, Memphis, TN 38105, USA; 3 Department of Pathology, P.J. Safárik University, Faculty of Medicine, Kosice 04001, Slovak Republic; 4 Departments of Oncology and Clinical Neurosciences, University of Calgary, Calgary, Alberta, T2N 4N1, Canada

**Keywords:** Inflammation, myelin repair, NG2 ablation, oligodendrocyte progenitors, pericytes, macrophages

## Abstract

**Background:**

Multiple sclerosis (MS) is a demyelinating disease in which blood-derived immune cells and activated microglia damage myelin in the central nervous system. While oligodendrocyte progenitor cells (OPCs) are essential for generating oligodendrocytes for myelin repair, other cell types also participate in the damage and repair processes. The NG2 proteoglycan is expressed by OPCs, pericytes, and macrophages/microglia. In this report we investigate the effects of NG2 on these cell types during spinal cord demyelination/remyelination.

**Methods:**

Demyelinated lesions were created by microinjecting 1% lysolecithin into the lumbar spinal cord. Following demyelination, NG2 expression patterns in wild type mice were studied via immunostaining. Immunolabeling was also used in wild type and NG2 null mice to compare the extent of myelin damage, the kinetics of myelin repair, and the respective responses of OPCs, pericytes, and macrophages/microglia. Cell proliferation was quantified by studies of BrdU incorporation, and cytokine expression levels were evaluated using qRT-PCR.

**Results:**

The initial volume of spinal cord demyelination in wild type mice is twice as large as in NG2 null mice. However, over the ensuing 5 weeks there is a 6-fold improvement in myelination in wild type mice, versus only a 2-fold improvement in NG2 null mice. NG2 ablation also results in reduced numbers of each of the three affected cell types. BrdU incorporation studies reveal that reduced cell proliferation is an important factor underlying NG2-dependent decreases in each of the three key cell populations. In addition, NG2 ablation reduces macrophage/microglial cell migration and shifts cytokine expression from a pro-inflammatory to anti-inflammatory phenotype.

**Conclusions:**

Loss of NG2 expression leads to decreased proliferation of OPCs, pericytes, and macrophages/microglia, reducing the abundance of all three cell types in demyelinated spinal cord lesions. As a result of these NG2-dependent changes, the course of demyelination and remyelination in NG2 null mice differs from that seen in wild type mice, with both myelin damage and repair being reduced in the NG2 null mouse. These studies identify NG2 as an important factor in regulating myelin processing, suggesting that therapeutic targeting of the proteoglycan might offer a means of manipulating cell behavior in demyelinating diseases.

## Background

During the acute phase of multiple sclerosis (MS), damage to the blood-brain barrier allows infiltration of blood-derived cells that cause disruption of the myelin sheath [1-5]. The capability of the CNS for myelin repair is mediated by the action of oligodendrocyte progenitor cells (OPCs), which not only generate oligodendrocytes during CNS development, but also persist as the largest cycling population in the mature CNS [6-9]. These "adult" OPCs serve as a source of cells for myelin repair [8,10-12], but also exhibit other functions of mature glia [13], including contributions to nodes of Ranvier [14-16] and reception of synaptic input [17,18]. OPC function and remyelination of axons nevertheless often fail in both relapsing-remitting and progressive MS [19-21]. The inability of OPCs to produce adequate numbers of myelinating oligodendrocytes has been attributed to several factors, including failure of OPC proliferation, failure of OPC recruitment to the lesion, failure of OPC differentiation, and failure of OPCs or oligodendrocytes to interact with neurons. Compounding this complexity, MS is a multifactorial disease, involving participation of multiple factors in both myelin damage and myelin repair. A better understanding of the molecular mechanisms of myelin degradation and regeneration is clearly required for improved treatment of this primary demyelinating disease.

Here we show that the NG2 proteoglycan is expressed by three cell types that invade demyelinated CNS lesions: OPCs, macrophages/microglia, and microvascular pericytes. In addition to serving as a marker for these cell types [22,23], NG2 also promotes cell proliferation and motility. In the neonatal NG2 null mouse, decreased OPC proliferation reduces the pool of progenitors available for generating myelinating oligodendrocytes, resulting in reduced developmental myelination in the cerebellum [24]. Ablation of NG2 also causes deficits in pericyte function. Decreased pericyte recruitment and interaction with endothelial cells lead to diminished vascularization in both ocular and tumor models in the NG2 null mouse [25,26]. We therefore have the ability to investigate the role of NG2 in multiple cell types during the processes of demyelination and remyelination.

Following microinjection of L-α-lysolecithin into the spinal cord white matter, we have investigated the activation, proliferation, recruitment, and maturation of cells that are normally NG2-positive in the wild type mouse. The importance of the NG2 molecule and NG2-positive cells in demyelination and remyelination has been evaluated via comparisons of wild type and NG2 knockout animals. The absence of NG2 causes significant deficits in the behavior of OPCs, macrophages/microglia, and pericytes, accompanied by quantitative changes in the phenomena associated with axon demyelination and remyelination.

## Methods

### Animals

Animal work was performed according to guidelines issued by the National Institutes of Health, following procedures approved by the Office of Laboratory Animal Welfare. All experimental protocols were approved by the Sanford-Burnham Institutional Animal Care and Use Committee. The current experiments utilized male wild type (NG2+/+) and NG2 null (NG2-/-) mice between the ages of 3-5 months. NG2 null mice were generated by a homologous recombination strategy and backcrossed for 10 generations onto the C57Bl/6 background [27].

### Lysolecithin-induced demyelination in the spinal cord of mice

For spinal cord surgery, male mice (28-38 g) were anesthetized with Ketamine/Xylazine (100/10 mg/kg) administered intraperitoneally. Depth of anesthesia was assured by monitoring lack of response to a noxious foot pinch prior to commencing surgery. A skin incision was made above the lower thoracic vertebrae. Paravertebral muscles on both sides of the Th_11_-L_1 _vertebrae were cut, and the vertebral column was stabilized with transverse process clamps (Stoelting). The spinal cord was exposed between the Th_12_-Th_13 _vertebrae, and a small incision was made in the dura just lateral to the posterior spinal vein. A 1.5 μl solution of 1% L-α-lysolecithin (Lysophosphatidylcholine; Sigma, St. Louis, MO) in 0.1 M phosphate buffer was injected 0.5 mm deep into the dorsal column at a rate of 0.75 μl/minute. This was accomplished using a micromanipulator (Stoelting, Wood Dale, IL), 32 G needle, 5 μl syringe (7762-05, 87930; Hamilton), and digital injector (Harvard Apparatus, Holliston, MA). As a sham control, injections were done with 0.1 M PBS. The needle was left in place for an additional 2 min to avoid backflow of the lysolecithin or PBS. The muscle and skin incisions were sutured with gut and nylon, respectively (Harvard apparatus). In order to reduce postoperative pain after recovery from anesthesia, animals received a subcutaneous injection of buprenorphine (1.0 mg/kg).

### Tissue preparation and immunocytochemistry

Some animals received intraperitoneal doses of 5-bromo-2-deoxyuridine (BrdU, 80 mg/kg) on post-surgery day 4, three days prior to euthanasia at day 7. At 1, 2, and 6 weeks after lysolecithin injection, animals were deeply anesthetized with Ketamine/Xylazine (100/10 mg/kg) and transcardially perfused with 0.1 M PBS, followed by 4% paraformaldehyde (pH 7.4). Spinal cords were removed and post-fixed for 24 hours at 4°C in the same fixative used for transcardial perfusion. Spinal cords were cryoprotected for 24 hours at 4°C in 0.1 M phosphate buffer containing 20% sucrose. Transverse sections (30 μm) were cut at -16°C on a cryostat microtome (Cryocut, 1800), and collected free-floating in 0.1 M PBS containing 0.02% sodium azide.

For immunostaining, free-floating sections were first incubated for 60 min at room temperature in 0.1 M PBS containing 5% normal goat serum and 0.5% Triton X-100. Sections were then incubated overnight at 4°C with primary antibodies diluted in PBS containing 0.8% Triton X-100, 0.02% sodium azide, and 5% normal goat serum. The following primary antibodies were used: 1) guinea pig anti-NG2 (1:25; [28]); 2) rabbit anti-PDGFRα (1:100; [29]); 3) rat anti-BrdU (OBT0030G, Serotec, 1:50); 4) mouse anti-Pan-Axonal Neurofilament (smi-312R, Sternberger, 1:500); 5) mouse or rabbit anti-myelin basic protein (MBP, Sternberger MSMI 94, 1:500 or Chemicon, AB980 1:100); 6) rabbit anti-PDGFRβ (1:100; [28]); 7) rat anti-mouse CD11b (550282, BD Pharmingen); 8) rabbit anti-IBA-1 (019-19741, Wako). After three 10-min washes with PBS, the sections were incubated with appropriate combinations of secondary antibodies: goat anti-mouse (Alexa 488; A11029, Invitrogen), anti-rabbit (Alexa 568; A11036 or Alexa 647; A21245, Invitrogen), donkey anti-guinea pig (Cy2 or Cy3; 706-225-148 or 706-165-148, Jackson ImmunoResearch), and/or goat anti-rat (Alexa 488; A11006, Invitrogen). Secondary antibodies were diluted 1:250 in the same solution as the primary antisera. In the case of BrdU, sections were incubated in 2N HCl for 30 min at 37°C, followed by boric acid neutralization (pH 8.5) for 10 min, and then processed via the immunostaining protocol described above. 4'-6-diamidino-2-phenylindole (DAPI, 4 μg/mL, D3571, Invitrogen) was used for general nuclear staining of all sections. After washing three times for 10 min with PBS, the sections were mounted on slides, air-dried, and then cover-slipped with Vectashield (H-1000, Vector lab).

In some cases myelin was also visualized histochemically in 5 μm thick paraffin sections using Kiernan's Eriochrome Cyanin technique [30], coupled with counterstaining by Nuclear Fast Red (H-3403, Vector lab).

### Quantitative RT-PCR analysis

For quantitative RT-PCR analysis, 6 mice of each genotype at 7 days postsurgery were deeply anesthetized with Ketamine/Xylazine and rapidly decapitated. Spinal cords removed by hydroextrusion were immersed in RNA stabilization reagent (76104, Qiagen), and 6 mm segments were dissected, spanning from 3 mm above to 3 mm below the lysolecithin injection site. Dissected spinal cord segments were immersed for 30 seconds in isopentane on dry ice and then stored at -80°C. For RNA isolation, the frozen spinal cords were homogenized in liquid nitrogen, and total RNA was isolated using an RNeasy^® ^Lipid Tissue Mini Kit (# 74804, Qiagen) following the manufacturer's instructions. Complementary DNA was prepared from 1-2.5 μg of total RNA from each sample using the Superscript^® ^First-Strand RT-PCR kit (# 11904018, Invitrogen). Diluted cDNA aliquots were then used for 20 μl PCR reactions with Brilliant^® ^II SYBR^® ^Green qPCR Master Mix (Stratagene) and appropriate primers at concentrations of 200 nM each. PCR reactions were run in duplicate for each primer pair, and transcripts were quantified in the MXP 3000 qPCR System (Stratagene). Transcript levels were normalized to expression of mRNA for the housekeeping gene glyceraldehyde-3-phosphate dehydrogenase (GAPDH), and normalized expression levels for each test gene in the NG2 null mouse were compared to levels found in wild type mice, which were defined as being equal to 1. Following qRT-PCR, the identity of RT-PCR products was confirmed by agarose gel electrophoresis. Sequences of oligonucleotide primers used in this study are shown in the Table 1.

**Table 1:** Primer sequences used in qRT-PCR

Gene	Primer Sequence
GAPDH forward	5'-CCA GTA TGA CTC CAC TCA CG-3'
GAPDH reverse	5'-GAC TCC ACG ACA TAC TCA GC-3'
IFNγ forward	5'-TGC TGA TGG GAG GAG ATG TCT-3';
IFNγ reverse	5'-TTT CTT TCA GGG ACA GCC TGT T-3';
IL-4 forward	5'-AGG TCA CAG GAG AAG GGA CGC C-3'
IL-4 reverse	5'-TGC GAA GCA CCT TGG AAG CCC-3'
IL-10 forward	5'-CTG GAC AAC ATA CTG CTA ACC G-3'
IL-10 reverse	5'-GGG CAT CAC TTC TAC CAG GTA A-3'
IL-1β forward	5'-GCC CAT CCT CTG TGA CTC AT-3'
IL-1β reverse	5'-AGG CCA CAG GTA TTT TGT CG-3'

### Image processing and quantification

At least 4 wild type and 4 NG2 null male mice were examined at each time point for quantitative analyses of various aspects of demyelination and remyelination. For calculation of demyelination volume, every 10^th ^section from a 6 mm segment of spinal cord (i.e., a total of twenty 30 μm sections spanning from 3 mm above to 3 mm below the injection site) was immunostained for MBP. A Nikon fluorescence microscope was used to acquire images of each section, allowing determination of individual areas of demyelination (mm^2^) via image analysis (Image Pro Plus 5.1; Media Cybernetics). Each individual value was multiplied by 10 to obtain the demyelinated volume for that particular segment of 10 sections, and all 20 values were then summed to obtain the total volume of demyelination. For animals of the same genotype and survival period, an average volume of demyelination was obtained and expressed as a mean value ± SD.

The location and abundance of PDGFRα, PDGFRβ, and CD11b immunoreactive cells in the dorsal column were analyzed in 7 sections spanning 1 mm of the central part of the demyelinated lesion. Immunostained sections were scanned via confocal microscopy (FV 1000 and FV10-ASW Ver. 2.0, Olympus). From each scan, we assembled a z-stack of 11 optical sections, each separated by 1 μm. Data from each of the z-stacks were averaged to yield values for the density of immunoreactive cells.

Colocalization of PDGFRα, PDGFRβ, CD11b, or IBA-1 immunoreactivity with immunostaining for either NG2 or BrdU was analyzed in a single optical section obtained from each of 7 sections. For these double labeling studies, the threshold for image capture was set high enough to avoid low levels of diffuse staining due to the presence of proteolytically shed NG2. This allowed us to focus on localization of cell surface NG2. Mitotic indices for PDGFRα, PDGFRβ and IBA-1 immunoreactive cells were calculated as the percentage of BrdU-positive cells in each of the three cellular populations.

Throughout the various analyses, images were processed with Adobe Photoshop CS3 Ver. 10.0 (Adobe Systems) to standardize brightness and contrast. All data were analyzed statistically using ANOVA and un-paired t-tests. P-values less than 0.05 were considered statistically significant.

## Results

### NG2 expression in wild type animals following lysolecithin injection

**Figure 1:**
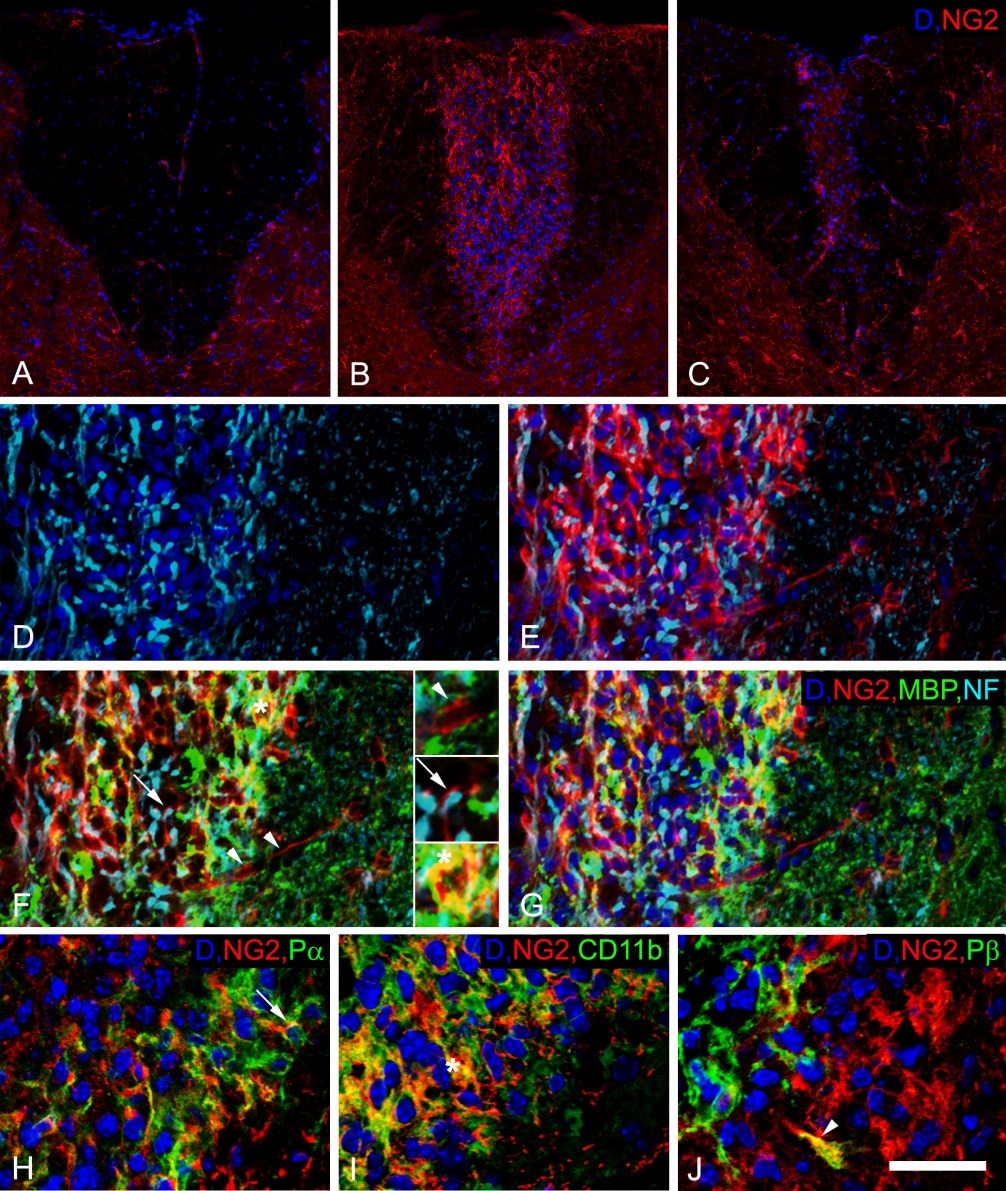
**NG2 expression in the dorsal column of wild type animals following lysolecthin injection**. Compared to sham-operated animals (A), an increased number of dapi (blue) positive nuclei, as well as increased NG2 expression (red), are observed within the injury site at 1 week (B and D-J) and 6 weeks (C) after lysolecithin injection. Panels D-G depict labeling in the same tissue section with multiple markers. More intense expression of pan-neurofilament (NF, cyan) is seen in axons in the inflammatory region (D-G). NG2 (red) positive cells are seen (1) closely apposed to vessel-like structures (arrowheads), (2) in close proximity to completely or partially demyelinated axons (arrow), as judged by NF (cyan) and MBP (green) staining (F and G), and (3) in association with MBP labeling (asterisk, yellow, F). Each of these patterns of NG2 expression is magnified in the inset panels in F. Double labeling for NG2 along with PDGFR alpha (arrow, Pα, H), CD11b (asterisk, I), or PDGFR beta (arrowhead, Pβ, J) reveals NG2 co-expression by oligodendrocyte progenitors (H), macrophages/microglial cells (I), or pericytes/mesenchymal stem cells (J). Scale bar = 100 μm (A-C), 50 μm (D-G), and 30 μm (H-J).

Compared to sham-operated animals injected with 1.5 μL of PBS (Figure 1A), wild type mice injected with lysolecithin exhibited increased NG2 expression in the damaged region of the spinal cord (Figure 1B, C). The greatest increase in NG2 expression was detected 1 week after lysolecithin injection (Figure 1B and Table 2). At the injury site one week after lysolecithin injection, we also detected more than a 3-fold increase in cell density compared to the dorsal columns of sham-operated mice. In Figures 1D-G, invading cells are present at sites of axonal demyelination, visualized by antibodies against neurofilament protein (NF) and myelin basic protein (MBP). NG2-positive cells are seen in close proximity to completely or partially (arrow) demyelinated axons (Figure 1F) and in association with vessel-like structures (arrowheads). MBP was observed within some NG2-immunoreactive cells (Figure 1F, asterisk), possibly indicative of phagocytosis by macrophages. Double immunolabeling shows that NG2 is expressed by platelet-derived growth factor receptor alpha-positive OPCs (Figure 1H, arrow), CD11b-positive macrophages/microglial cells (Figure 1I, asterisk), and PDGFRβ-positive pericytes (Figure 1J, arrowhead) in the inflammatory region. For these studies, CD11b was chosen over other macrophage/microglial markers because of its expression on a relatively high percentage of NG2-positive cells.

**Table 2:** Abundance of NG2, PDGFR alpha, CD11b, and PDGFR beta expressing cells in wild type and NG2 null mice 1, 2, and 6 weeks after lysolecithin injection.

		*NG2 (%)*	*PDGFRα (%)*	*CD11b (%)*	*PDGFRβ (%)*
*Sham*	*WT*	6.12 ± 1.7^c^	8.32 ± 2.1^c^	-	6.62 ± 0.9^c^
	*KO*	-	6.77 ± 2.6^c^	-	4.82 ± 1.4^c^
*1W*	*WT*	100 ± 8.2	100 ± 11.5	100 ± 9.1	100 ± 17.7
	*KO*	-	72.69 ± 16.8*	33.23 ± 10.3***	60.59 ± 15*
*2W*	*WT*	75.45 ± 6^b^	111.4 ± 1.9	174.83 ± 18.6^c^	121.71 ± 18.7
	*KO*	-	89.7 ± 6.6***	103.13 ± 16.6^c^**	87.71 ± 12.4^a^*
*6W*	*WT*	23.99 ± 11.7^c^	33.01 ± 4.9^c^	6.41 ± 0.8^c^	33.83 ± 2.6^c^
	*KO*	-	40.8 ± 6.4^a^	4.21 ± 1.3^c^*	44.69 ± 7*

The abundance of various cell types (NG2+, PDGFRα+, CD11b+, PDGFRβ+) in demyelinated lesions from 1-6 weeks post-lysolecithin injection is illustrated by normalizing cell density values to cell densities found in wild type mice at 1 week post-surgery (these 1 week values are designated as 100%). Values represent means ± S.D. Statistically significant differences are indicated by * < 0.05; ** < 0.01; *** < 0.001 when values were compared between WT and NG2 null mice at the same post-injection week. ^a ^< 0.05; ^b ^< 0.01; ^c ^< 0.001 represent statistically significant differences between values obtained for mice of the same genotype when compared to the 1^st ^week post-injection.

### Lysolecithin-induced demyelination in wild type and NG2 null mice

**Figure 2:**
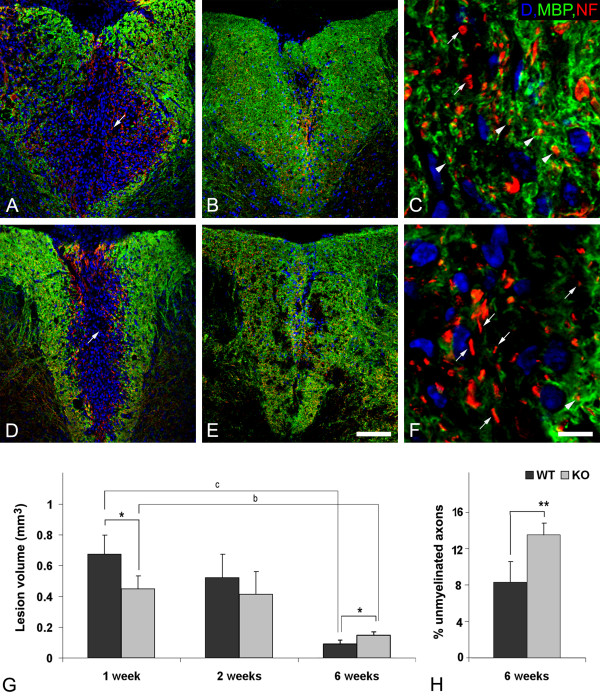
**Demyelination and remyelination in dorsal columns of wild type (WT) and NG2 null (KO) mice**. Immunolabeling for MBP (green) and neurofilament (NF, red) reveals greater initial demyelination in wild type (A) compared to NG2 null spinal cord (D) during the first post-surgery week. However, better repair is seen in wild type (B and C) than in knockout (E and F) spinal cord at 6 weeks after surgery. The higher resolution images in C and F allow identification of NF-positive axons (red) associated with (arrowheads) or lacking association with (arrows) MBP-positive myelin (green) at 6 weeks post-injury. Quantification of white matter lesion volumes, defined as MBP-negative regions (see panels A, B, D and E), in wild type and NG2 null mice reveals larger lesions in wild type mice one week after lysolecithin injection, but diminished repair of lesions in NG2 null mice six weeks post-injury. Lesion volumes are expressed as mean values ± SD. (G). An increased number of demyelinated axons (H), determined by MBP and NF double labeling (see panels C and F), were present in the dorsal column of NG2 null mice 6 weeks after lysolecithin injection. Statistically significant differences are indicated by * < 0.05; ** < 0.01 when values for WT and KO mice are compared at the same time point; ^b ^< 0.01; ^c ^< 0.001 indicate statistically significant differences within the same genotype at 1 and 6 weeks after lysolecithin injection. Scale bar = 100 μm (A, B, D and E) and 8 μm (C and F).

Use of MBP staining (green) to compare demyelinated regions in the white matter of wild type and NG2 null mouse spinal cords one week after lysolecithin injection reveals a 43.67 ± 11.54% decrease in injury volume in the absence of NG2 (Figures 2A, D and 2G). However, over the ensuing 5 weeks, only a small degree of damage repair is seen in NG2 null mice (Figures 2E and 2F), while a marked improvement is observed in wild type mice (Figures 2B and 2C). At 6 weeks post-injection, a 6-fold repair of myelin is found in the wild type mice, compared to only a 2-fold recovery in NG2 null mice (Figure 2G). Qualitatively similar results were obtained using eriochrome cyanin staining to quantify the extent of myelin damage (data not shown). Thus, despite the initially larger extent of inflammation and loss of myelin in wild type mice, myelin repair is superior in these mice to the recovery observed in NG2 null mice. We used double immunostaining for MBP (green) and NF (red) to evaluate the extent to which axons were remyelinated in the two sets of mice at 6 weeks post-injection (Figures 2C and 2F). Quantification of NF-positive axons tightly associated with MBP revealed that more dorsal column axons remained unmyelinated in the absence of NG2 (Figure 2H).

### Effects of NG2 ablation on abundance of specific cell types during demyelination and remyelination

Along with comparisons of demyelination and remyelination in wild type and NG2 null mice, we evaluated the recruitment and abundance of specific cell types during the injury and repair processes. We focused on OPCs, macrophages/microglial cells, and pericytes; i.e. the cells in wild type mice that express NG2 under physiological or pathological conditions.

**Figure 3:**
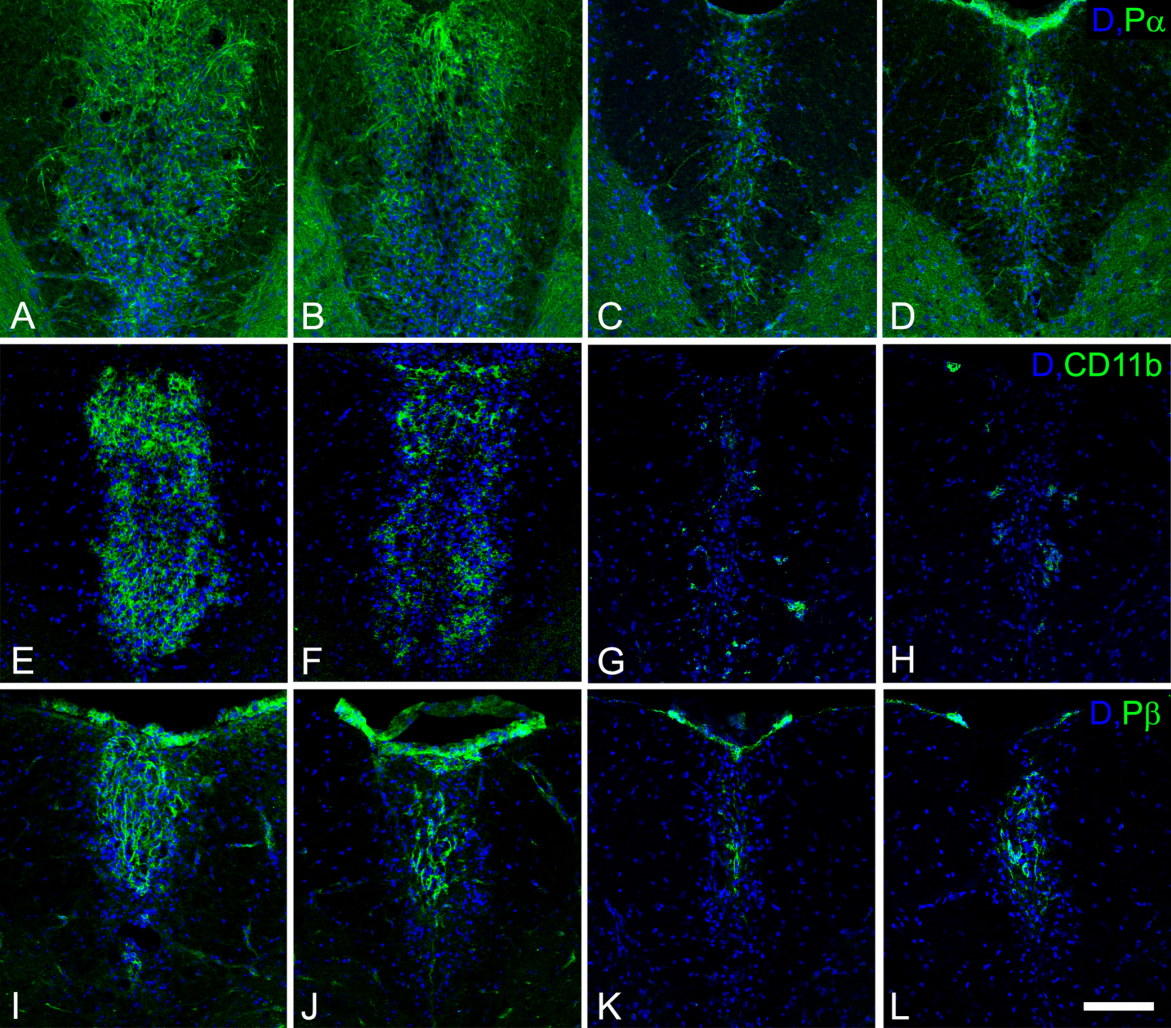
**Distribution of PDGFR alpha, CD11b, and PDGFR beta immunoreactive cells in injured spinal cord white matter**. Panels A-D show the distribution of PDGFR alpha positive OPCs (green) at 1 (A, B) and 6 (C, D) weeks after demyelination insult. Panels E-H present CD11b immunoreactive myeloid cells (green) at 1 (E, F) and 6 weeks (G, H) post-injury, while panels I-L show PDGFR beta positive cells (green) at 1 (I, J) and 6 weeks (K, L) post-injury. The first and third columns show sections from wild type mice at 1 and 6 weeks, respectively, after demyelination insult. The second and fourth columns show sections from NG2 null mice at 1 and 6 weeks, respectively, after demyelination insult. Blue: DAPI. Scale bar = 100 μm.

The abundance of OPCs, macrophages/microglial cells, and pericytes in demyelinated lesions was determined by immunostaining for PDGFRα, CD11b or IBA-1, and PDGFRβ, respectively, and positive areas of immunoreactivity in the dorsal column of the spinal cord were quantified by image analysis. The dorsal columns of uninjured wild type and NG2 null mice did not exhibit statistically significant differences in the numbers of PDGFRα-positive OPCs or PDGFRβ-positive pericytes, although there was a trend toward lower numbers in the NG2 null mouse in both cases (Table 2). However, one week after lysolecithin injection, lesion sites in wild type and NG2 null mice contained significantly different numbers of these cell types. Compared to wild type animals, lesions in NG2 null mice contained almost 30% fewer OPCs than lesions in wild type mice (Figures 3A, B and Table 2). This same trend was also observed in the cases of macrophages/microglial cells (Figures 3E and 3F) and pericytes (Figures 3I and 3J). The most remarkable difference was found in the case of macrophages/microglial cells, where approximately 3-fold fewer CD11b-positive macrophages/microglial cells were found in NG2 null mice than in wild type animals (Table 2).

Six weeks after lysolecithin injection, CD11b-positive macrophages/microglial cells are still seen less frequently in NG2 null lesions than in wild type lesions (Figures 3G and 3H). However, PDGFα-positive OPCs (Figures 3C and 3D) and PDGFRβ-positive pericytes (Figures 3K and 3L) now appear to be more abundant in NG2 null lesions than in wild type lesions. We believe this is due to delayed recruitment of immature OPCs and pericytes in the absence of NG2. In wild type animals, maturing cells recruited at earlier time points may have already down-regulated expression of the PDGFRα and PDGFRβ markers.

### Effect of NG2 ablation on cytokine expression

**Figure 4:**
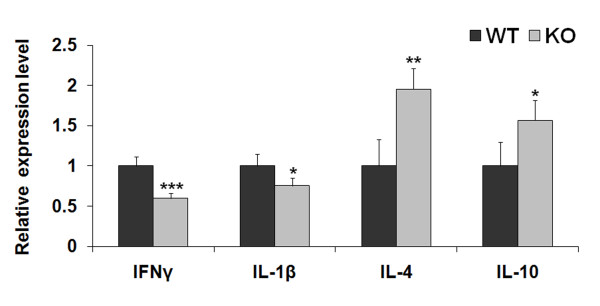
**Relative expression levels of IFNγ, IL-1β, IL-4, and IL-10 transcripts 7 days after lysolecithin injection**. Cytokine levels in NG2 null mice were normalized to those seen in wild type mice, defined as being equal to 1. Relative cytokine expression levels are expressed as mean values ± SD. Statistically significant differences between WT and KO values are indicated by * < 0.05, ** < 0.01, and *** < 0.001.

In addition to reduced influx of CD11b-immunoreactive macrophages/microglial cells into the damaged white matter one week after lysolecithin injection into NG2 null mice, we also observed changes in cytokine levels indicative of a shift from a pro-inflammatory to anti-inflammatory phenotype [31]. Analysis of transcript levels by qRT-PCR revealed that transcripts for the pro-inflammatory cytokines interferon gamma (IFNγ) and interleukin 1-beta (IL-1β) were reduced in NG2 null mice. In contrast, the expression of cytokines characteristic of an anti-inflammatory phenotype (IL-4 and IL-10) was increased by ablation of NG2 (Figure 4).

### Effects of NG2 ablation on cell proliferation and motility

**Figure 5:**
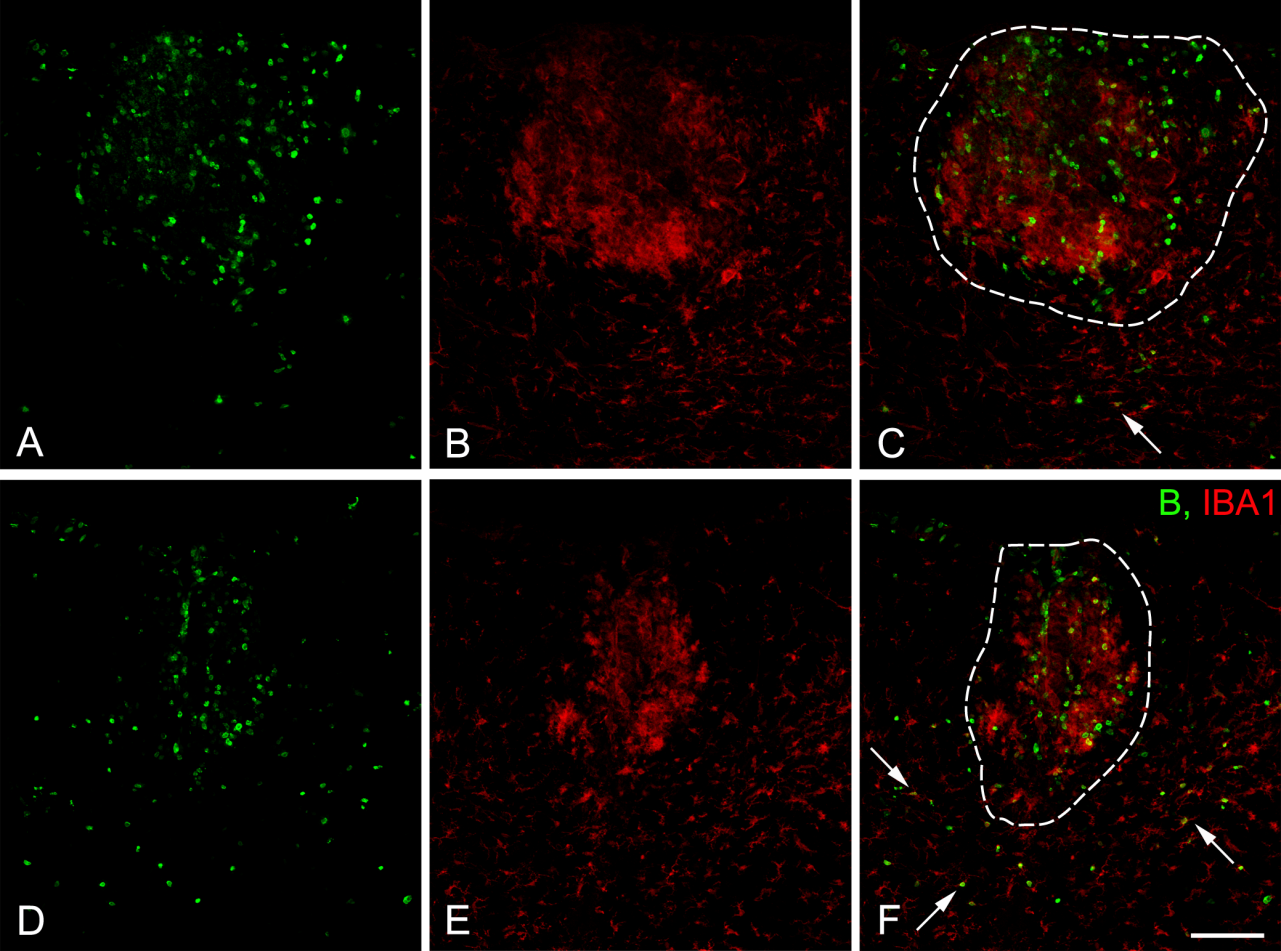
**Proliferation of IBA-1 immunoreactive macrophages/microglial cells in the injured spinal cord**. Sections of injured spinal cord were evaluated for BrdU incorporation (green) and IBA-1 labeling (red) 7 days after injury (3 days after BrdU injection). Compared to wild type animals (A-C), fewer proliferating IBA-1-positive cells are seen in NG2 null mice (D-F). Boundaries of demyelinated lesions are indicated by white dotted lines in C and F. In NG2 null mice, IBA-1/BrdU double-labeled cells (arrows) remain outside the lesion to a greater extent than in wild type mice. Scale bar = 100 μm.

Proliferation of OPCs, pericytes, and macrophages/microglial cells in demyelinated lesions in wild type and NG2 null mice was evaluated by BrdU incorporation. BrdU was injected 4 days after surgery and animals were euthanized after an additional 3 days (i.e. at day 7). We found that the mitotic indices of OPCs, pericytes, and macrophages/microglia were all reduced in the absence of NG2 (Table 3). While OPCs proliferated in proximity to demyelinated axons inside the lesion site, some BrdU-positive macrophages/microglial cells were also seen outside the lesion (Figure 5). For these studies we used the IBA-1 marker because of its expression on both resident microglia and infiltrating macrophages/microglial cells, thus allowing us to assess proliferation in both populations. The presence of extra-lesional BrdU-labeled IBA-1-positive cells suggested the possibility that microglial cells generated outside the demyelinated region might invade the lesion, contributing to the pool of inflammatory cells present in this area. To examine this possibility, we examined BrdU incorporation after a one-day incubation period. BrdU was administered at 4 days after lysolecithin injection, and animals were euthanized on the following day. Table 4 shows that in both wild type and NG2 null mice, about 10% of IBA-1, BrdU-double positive cells were located outside the demyelinated area on this first day. However by day 3, only 1% of these cells were still outside the lesion in wild type mice, whereas at least 7% of the cells in NG2 null mice were still located external to the lesion. This result indicates a possible role for NG2 in the motility of macrophages/microglia.

**Table 3:** Proliferation of PDGFRα, PDGFRβ, and IBA-1 expressing cells in wild type and NG2 null mice.

	WT	KO
*Total Pα positive cells*	178.23 ± 20.3	127.94 ± 18.4*
*Pα/BrdU positive cells*	26.4 ± 2.2	10.76 ± 2.9
*Mitotic indices*	14.81 ± 3.1%	8.41 ± 0.6%**
*Total Pβ positive cells*	29.8 ± 5.7	21.91 ± 3.4*
*Pβ/BrdU positive cells*	4.53 ± 0.8	2.31 ± 0.9
*Mitotic indices*	15.2 ± 0.6%	10.52 ± 0.6%***
*Total IBA-1 positive cells*	217.94 ± 14.8	74.45 ± 12.3***
*IBA1/BrdU positive cells*	19.66 ± 3.6	4.71 ± 3.5
*Mitotic indices*	9.02 ± 1.2%	6.32 ± 1.8%*

Total numbers of PDGFRα (Pα), PDGFRβ (Pβ), and IBA-1 positive cells, along with BrdU incorporation, were determined in 0.1 mm^2 ^areas of the dorsal column at 7 days postsurgery. Mitotic labeling indices for OPCs, pericytes, and macrophages/microglial cells are expressed as the percentage of each cell type that is BrdU positive. Data represent the mean ± S.D. Statistically significant differences between wild type and NG2 null mice are indicated by * < 0.05; ** < 0.01; *** < 0.001.

**Table 4:** Percentage of IBA-1/BrdU-positive cells outside the demyelinated region.

	WT	KO
*5^th ^postsurgery day*	7.74 ± 0.44%	10.29 ± 0.31%
*7^th ^postsurgery day*	1.12 ± 0.01% ^a^	7.22 ± 0.12% ***

The percentage of BrdU-labeled cells IBA-1-positive cells outside the area of demyelination was evaluated in wild type (WT) and NG2 null (KO) mice at 5 and 7 days following lysolecithin injection. Data represent the mean ± S.D. A statistically significant difference between wild type and NG2 null mice at day 7 is indicated by *** < 0.001. ^a ^< 0.05 indicates the statistically significant difference in wild type mice between the 5^th ^and 7^th ^post-injection days.

## Discussion

In the CNS, myelination is accomplished by mature oligodendrocytes that arise from OPCs. During CNS development, a substantial pool of OPCs must be generated for production of mature oligodendrocytes in sufficient numbers for adequate myelination of axons. The adult CNS still contains large numbers of OPCs that differ somewhat from perinatal progenitors in their capability for motility and proliferation, yet respond to most of the same stimuli and express a similar set of phenotypic markers as their perinatal counterparts. Adult OPCs account for a large percentage of the proliferating cells in the mature CNS [7,9] and are responsible for production of new oligodendrocytes to replace damaged cells. Newly-differentiated oligodendrocytes derived from adult OPCs, rather than pre-existing oligodendrocytes, are responsible for remyelination of axons that occurs following various types of demyelinating events [8,10,32-34]. Factors that influence OPC proliferation and differentiation are therefore of great importance for our understanding of both developmental myelination and myelin repair.

The NG2 proteoglycan contributes to the proliferation of OPCs during CNS development. In the NG2 null mouse, decreased OPC proliferation reduces the size of the OPC pool, leading to a delay in production of normal numbers of mature oligodendrocytes and to a corresponding delay in axon myelination [24]. We have used lysolecithin-induced demyelination of the spinal cord to examine the possibility that ablation of NG2 also impedes repair of myelin damage in the adult CNS. Following microinjection into CNS white matter, lysolecithin replaces phospholipids and forms micelles in the membrane bilayer [35], rapidly inducing local myelin destruction [36], blood-brain barrier damage, and recruitment of macrophages and local microglial cells into the lesion site [4]. This commonly-used demyelination model [4,19,35-37] has the advantage that the site and extent of the injury are well-defined and reproducible, facilitating data acquisition. In addition, lysolecithin-induced demyelination occurs as an acute event, such that all subsequent phenomena are associated with the regenerative response. This provides a useful means of separating events and mechanisms associated with the respective processes of demyelination and remyelination [21].

The regeneration of myelin following demyelination is a multifactorial process, due in part to the involvement of multiple cell types in the damage and repair mechanisms. In addition to neurons and OPCs, microglia, macrophages, and pericytes also contribute to these processes [38-41]. Our work shows that the NG2 proteoglycan is expressed by three cell types that invade demyelinated lesions: OPCs, pericytes, and macrophages/microglia. The differential contributions of these three cell types to the damage and repair processes, combined with differences in NG2 function in the respective cell types, are probably responsible for the complex patterns of demyelination and remyelination that we see in the global NG2 null mouse. Figure 2 shows that although the extent of initial demyelination is reduced in the NG2 null mouse, repair of this lesion nevertheless proceeds more slowly than repair of the larger lesion found in the wild type mouse. The impact of NG2 ablation on OPCs is likely confined to deficiencies seen during the repair process, since OPCs generate oligodendrocytes that carry out remyelination. Conversely, diminished involvement of macrophages/microglia probably provides the best explanation for the reduced extent of initial demyelination seen in the NG2 null mouse. However, macrophages/microglial cells also contribute to myelin repair by clearing myelin debris and by producing cytokines and growth factors that promote recruitment of OPCs and prime interactions between OPCs and axons. Thus, NG2-dependent deficits in macrophage/microglia function may also contribute to the reduced myelin repair seen in the NG2 null mouse. Similarly, it is possible that pericytes affect both myelin damage and repair. The recruitment of pericytes for revascularization of the lesion and repair of the blood brain barrier likely plays an important role in the healing process. However, vascularization also provides increased access to inflammatory cells and cytokines that contribute to myelin damage [40,42-45]. Since many of the pericytes in lysolecithin-induced lesions are not associated with vascular endothelial cells, another consideration is the ability of pericytes to serve as mesenchymal stem cells [46,47] with immunomodulatory properties that can promote myelin repair via their effects on the activities of inflammatory cells [48].

Our evidence suggests that promoting cell proliferation is a key functional role for NG2 in OPCs, pericytes, and macrophages/microglia. BrdU incorporation reveals significant reductions in mitotic index for all three cell types in demyelinated lesions in the NG2 null mouse. In the case of OPCs, this confirms a similar result obtained in our studies of developmental myelination: namely, that ablation of NG2 reduces the OPC mitotic index, with a corresponding decrease in the number of myelinating oligodendrocytes [24]. Thus, NG2 is important for promoting the proliferation of both perinatal OPCs and adult OPCs. The BrdU results also confirm our report that ablation of NG2 diminishes pericyte proliferation during pathological retinal neovascularization, leading to decreased blood vessel formation in the retinas of NG2 null mice [25]. This negative effect of NG2 ablation on cell proliferation may be a fairly general one, since we also observe diminished keratinocyte proliferation in the skin of newborn NG2 null mice [49]. Our in vitro studies also support a role for NG2 in promoting cell proliferation. NG2 is able to enhance proliferation via two mechanisms: promotion of signaling by β1 integrins [50] and promotion of signaling by receptors for the growth factors PDGF and FGF [27,51].

In vitro studies also indicate that NG2-dependent signaling by β1 integrins and growth factor receptors can promote cell motility as well as cell proliferation [27,50,52,53]. In vivo, one indication of this effect is seen in our current studies on macrophage invasion into demyelinated lesions. BrdU tracking studies at day 5, one day after lysolecithin injection, show that 8 to 10% of the macrophages/microglia in dorsal column white matter are located outside demyelinated lesions. By 7 days post-injection in wild type mice, 90% of these peripherally-located cells have migrated into the lesion. By contrast, only 20% of extra-lesional cells have migrated into the lesion in NG2 null mice, indicative of the NG2 dependence of macrophage motility. Similar measurements were not possible in the case of OPCs or pericytes due to the rare occurrence of BrdU-labeled cells outside of demyelinated lesions.

Our finding of changes in cytokine expression following NG2 ablation may also be important in understanding changes in demyelination and remyelination in the NG2 null mouse. Although it remains to be determined whether changes in cytokine expression in the NG2 null mouse are associated with changes in macrophages as opposed to other inflammatory cell types, decreased levels of IFNγ and IL-1β coupled with increased levels of IL-4 and IL-10 suggest that NG2 ablation shifts a pro-inflammatory phenotype to an anti-inflammatory one. IFNγ provokes acute re-occurrence of demyelination in MS patients [54], and IL-1β is present in CNS-infiltrating myeloid cells in MS models [55]. It therefore seems possible that decreased levels of IFNγ and IL-1β in spinal cord lesions in the NG2 null mouse (or altered activities of cells expressing these cytokines) are responsible for the reduced white matter damage seen in these mice. Moreover, decreased IL-4 production in the CNS exacerbates experimental autoimmune encephalitis, and is associated with increased infiltration of inflammatory cells [56], while increased IL-10 expression is associated with reduced inflammation [57]. The possibility that NG2 null macrophages/microglia may exhibit less inflammatory properties than wild type cells is in line with the in vitro finding of a reduced inflammatory phenotype upon knockdown of NG2 in microglia [58]. We speculate that diminished occurrence of myeloid cells in NG2 null spinal cord lesions, coupled with alterations in the intrinsic properties/functions of NG2-negative macrophages/microglial cells, can affect the progression of demyelination and remyelination in NG2 null mice.

## Conclusions

In summary, our results demonstrate that the functions of all three of the NG2-positive cell types (OPCs, pericytes, and macrophages/microglia) associated with demyelinated lesions are compromised by the ablation of NG2. As a result of changes in multiple cell types, the respective processes of myelin damage and myelin repair are both altered in NG2 null mice. The complexity of the demyelination/remyelination phenotype in the global NG2 null mouse suggests that cell type-specific ablation of the proteoglycan will be a useful strategy for elucidating the respective contributions of NG2-positive cell types to the myelin damage and repair processes. The use of NG2 floxed mice in conjunction with appropriate Cre drivers will allow us to perform the desired NG2 ablations.

## List of abbreviations

BrdU: 5-bromo-2-deoxyuridine; CNS: central nervous system; DAPI: 4'-6-diamidino-2-phenylindole; GAPDH: glyceraldehyde-3-phosphate dehydrogenase; IFN: interferon; IL: interleukin; MS: Multiple sclerosis; MBP: myelin basic protein; NF: Pan-Axonal Neurofilament; NG2-/-: NG2 null mice; NG2+/+: wild type mice; OPCs: oligodendrocyte progenitor cells; PDGFR: Plate derived growth factor receptor; qRT-PCR: quantitative reverse transcription-polymerase chain reaction.

## Competing interests

The authors declare that they have no competing interests.

## Authors' contributions

WBS and KK designed and performed research, and prepared the manuscript. KK also evaluated the data. YC and AB performed research. VWY designed research. All authors have read and approved the final version of the manuscript.
